# Survey of current practices and opinions of German Society of Gynecologic Endoscopy members regarding the treatment of ovarian neoplasia by robotic surgery

**DOI:** 10.1007/s00404-020-05876-w

**Published:** 2020-11-17

**Authors:** J. S. M. Zimmermann, J. C. Radosa, M. P. Radosa, P. Sklavounos, P. A. Schweitzer, E. F. Solomayer

**Affiliations:** 1 Department of Gynecology and Obstetrics, Saarland University Hospital, Kirrbergerstraße 100, 66421 Homburg, Saar Germany; 2 Department of Gynecology and Obstetrics, Klinikum Bremen Nord, Bremen, Germany

**Keywords:** Minimally invasive surgery, Robotic surgery, Ovarian cancer, Borderline ovarian tumor, AGE

## Abstract

**Purpose:**

As data on this topic are sparse and contradictory, we aimed to ascertain the opinions of the members of the German Society of Gynecologic Endoscopy (AGE) regarding the use of robotic surgery in the treatment of ovarian malignancies.

**Methods:**

In 2015, an anonymous questionnaire was sent to AGE members to assess their views on the treatment of ovarian malignancies by robotic surgery according to T stage and the current treatment practices in their facilities.

**Results:**

Of the 228 respondents, 132 (58%) were fellows or attending physicians and 156 (68%) worked at university hospitals or tertiary referral centers. Most [*n* = 218 (96%)] respondents reported treating < 10% of their patients using robotic surgery. Respondents felt that T1 and borderline ovarian tumors, but not T2 (51%) or T3/4 (76%) tumors, should and could be treated by robot surgery. 162 (71%) respondents considered the currently available data on this subject to be insufficient, and 42% indicated their willingness to participate in clinical studies on the applicability of robotic surgery to the treatment of T1/2 ovarian tumors.

**Conclusion:**

The majority of AGE members surveyed considered robotic surgery to be an option for the treatment of T1 ovarian malignancies and borderline ovarian tumors. However, prospective randomized studies are needed to determine the relevance of robotic surgery in this context.

## Introduction

With approximately 7250 newly diagnosed cases per year, ovarian cancer is among the most frequently occurring cancers among women in Germany and the second leading cause of death from gynecological malignancies worldwide [1, 2]. Despite the optimization of chemotherapeutic regimens and the development of new therapies, surgery (using optimal procedures) remains the core element of ovarian cancer treatment. Survival rates depend directly on the extent of debulking, notably on the achievement of complete cytoreduction (R0 resection), and on the amount of tumor remaining in the abdomen postoperatively [3, 4].

Laparotomy has been the gold standard for the surgical treatment of gynecological malignancies. With constant progress in laparoscopy and robotic surgery in past decades and the widespread use of minimally invasive surgery (MIS) for the treatment of benign gynecological diseases, the focus has shifted gradually to the use of robotic surgery for the treatment of gynecological malignancies [5]. The benefits of MIS include reduced postoperative pain intensity, wound infection rates, length of hospitalization, and procedure-associated morbidity, as well as the possibility of immediate adjuvant therapy initiation. These advantages have led to the gradual implementation of laparoscopy and robotic surgery as alternatives to open surgery for the treatment of certain gynecologic malignancies [6]. Major concerns about MIS use in the gynecological context, however, are related to the ability to achieve sufficient oncological safety; they include the risks of intraoperative tumor rupture, port site metastasis, and peritoneal dissemination of tumor cells, as well as the questionable efficacy of surgical staging, which have prevented an implementation of laparoscopy and robotic surgery for the treatment of ovarian cancer [7–9].

The German S3 guidelines for diagnosis, treatment, and surveillance of ovarian malignancies recommend the use of MIS only in clinical trials, as few data regarding the oncological safety of these procedures are available and potential risks are not sufficiently recognized [10]. Internationally, some institutions have made efforts to implement MIS for the treatment of early-stage ovarian cancer, staging of advanced disease, and assessment of the neoadjuvant treatment response, and reported a comparable outcome of MIS in terms of feasibility and surgical parameters as against open surgery [11–13]. The role of MIS in the treatment of ovarian malignancies, however, remains controversial. This survey was conducted to assess the opinions of members of the German Society of Gynecologic Endoscopy (AGE) regarding the use of robotic surgery for the treatment of ovarian neoplasias and borderline tumors according to T stage, and to obtain information about current treatment practices in these members’ institutions.

## Methods

The present survey was the second part of a two-part study of the use of MIS in the treatment of ovarian neoplasia. The first survey examined the use of laparoscopy in the treatment of ovarian malignancies, and has been reported on elsewhere [14].

Before starting the study, AGE created a task force for questionnaire design, survey implementation, and data analysis. The study design was approved by the executive board of AGE [14]. According to the local ethics committee regulations (Saarland institutional review board) no ethical approval was needed for this survey. Informed consent for publication was obtained from all survey participants.

From February to October 2015, an anonymous online survey was sent to AGE members via email, and posted on the homepage of the AGE website. Two email reminders were sent during this period. The online survey was accessed via the Google Drive online survey system (Google Ireland Limited, Dublin, Ireland). After the data collection period, a research associate at the Department of Gynecology and Obstetrics, Saarland University Hospital, Homburg, entered the data without respondent-identifying information into an Excel (version 2010; Microsoft Corporation, Redmond, WA, USA) database.

The first part of the questionnaire solicited demographic and workplace information, such as respondents’ age, sex, and education level, as well as the hospital level and annual ovarian cancer surgery volume and respondents’ possible concerns regarding the oncological safety and precision of MIS for ovarian cancer in general. Those results have been published within the first manuscript on laparoscopic treatment of ovarian malignancies [14]. The second part solicited respondents’ opinions about the use of robotic surgery in the treatment of ovarian malignancies and borderline ovarian tumors, as well as information on current ovarian neoplasia treatment practices in their facilities. Respondents were asked about their opinions about the use of robotic surgery for ovarian neoplasia according to the T stage following the TNM classification for malignant tumors [15]. The next part of the survey solicited respondents’ opinions about currently available data on the use of robotic surgery in the treatment of ovarian neoplasia, and inquired about their willingness to participate in clinical trials on this topic. For the calculation of descriptive statistics, the data were transferred to SPSS (version 19; SPSS Inc., Chicago, IL, USA). Categorical data are reported as frequencies with percentages.

## Results

In total, 235 physicians who were AGE members (18% of those contacted) took part in the online survey. Seven incomplete questionnaires were excluded, leaving 228 completed questionnaires that were included in the final analysis.

### Respondent and practice characteristics

The sample comprised 128 (56%) male and 100 (44%) female gynecologists with a mean age of 36 (range, 26–62) years. 66 (29%) respondents were residents, 40 (18%) were fellows, 92 (40%) were attending physicians, and 30 (13%) were department heads. Almost half [*n* = 108 (47%)] of the respondents worked at university hospitals, 48 (21%) worked at clinics providing maximum or standard care, and 24 (11%) had private gynecological practices. One hundred twelve (49%) respondents indicated that they performed operations to treat ovarian malignancies and 172 (75%) indicated that they assisted with such interventions. 77 (34%) respondents reported that < 20 such operations were performed per year in their facilities, 106 (46%) reported 20–50 interventions per year, and 45 (20%) reported 50–100 interventions per year. Most [*n* = 218 (96%)] respondents indicated that robotic surgery was used in 0–10% of all ovarian cancer cases at their facilities (Table 1).

**Table 1:** Characteristics of participants (Radosa et al. [14])

	*N* (%)
Gender	
Male	128 (56%)
Female	100 (44%)
Age (years; median (range))	36 (26–62)
Level of training	
Resident	66 (29%)
Fellow	40 (18%)
Attending	92 (40%)
Head of department	30 (13%)
Hospital level	
University hospital	108 (47%)
Tertiary hospital	48 (21%)
Primary hosptial	48 (21%)
Day hospital	0 (0%)
Gynecological practice	24 (11%)
Do you perform surgery for treatment of ovarian malignancies yourself?	
Yes	112 (49%)
No	116 (51%)
Do you assist in surgery for treatment of ovarian malignancies?	
Yes	172 (75%)
No	56 (25%)
How many surgical procedures for primary ovarian malignancies are conducted at your institution per year?	
< 20	77 (34%)
20–50	106 (46%)
50–100	45 (20%)
> 100	0 (0%)
How many percent of these patients are treated via robotic surgery?	
0%	197 (87%)
< 10%	21 (9%)
10–40%	5 (2%)
> 50%	5 (2%)
Which concerns do you have regarding minimal invasive surgery treatment for ovarian malignancies?	
Danger of intraabdominal tumor cell dissemination	40 (18%)
Danger of port site metastasis	32 (14%)
Danger of rupture of ovarian mass	64 (27%)
Inaccuracy of peritoneal staging	68 (30%)
No disadvantage compared to open surgery	24 (11%)

### Current practice and perceived applicability of robotic surgery

42 (18%), 24 (10%), and 30 (13%) respondents reported that T1a, T1b, and T1c tumors, respectively, were treated with robotic surgery at their facilities; 164 (72%), 204 (90%), and 198 (87%) respondents reported no such practice for T1a–c tumors, respectively. 22 (10%) respondents reported using robotic surgery to treat T1a tumors in clinical trials; no respondent reported such use for T1b or T1c tumors. 14 (6%) respondents reported T2 tumor treatment with robotic surgery, and 214 (94%) respondents reported no such practice. Similarly, 216 (95%) respondents reported no use of robotic surgery to treat T3/4 ovarian tumors, and 12 (5%) respondents reported using such treatment only in clinical trials (Table 2).

**Table 2:** Current practice at participant institution for robotic treatment of ovarian cancer

	*N* (%)
What is the current practice at your institution (depending on T stage of disease)?	
T1a tumors	
Are treated via robotic surgery	42 (18%)
Are only treated as part of clinical trials via robotic surgery	22 (10%)
Are not treated via robotic surgery	164 (72%)
T1b tumors	
Are treated via robotic surgery	24 (10%)
Are only treated as part of clinical trials via robotic surgery	0 (0%)
Are not treated via robotic surgery	204 (90%)
T1c tumors	
Are treated via robotic surgery	30 (13%)
Are only treated as part of clinical trials via robotic surgery	0 (0%)
Are not treated via robotic surgery	198 (87%)
T2 tumors	
Are treated via robotic surgery	14 (6%)
Are only treated as part of clinical trials via robotic surgery	0 (0%)
Are not treated via robotic surgery	214 (94%)
T3/4 tumors	
Are treated via robotic surgery	0 (0%)
Are only treated as part of clinical trials via robotic surgery	12 (5%)
Are not treated via robotic surgery	216 (95%)

Reported concerns about the oncological safety of MIS in general were the inaccuracy of abdominal staging (30%) and the risks of ovarian tumor rupture (27%), intra-abdominal tumor cell spread (18%), and port-site metastasis (14%) [14]. 24 (11%) respondents indicated that they saw no disadvantage of the use of MIS relative to open surgery (Table 1) [14].

Approximately half of the respondents indicated that robotic surgery should or could be used to treat T1a [*n* = 136 (60%)], T1b [*n* = 108 (47%)], and T1c [*n* = 104 (46%)] tumors; 52 (22%), 60 (26%), and 57 (25%) respondents indicated that robotic surgery should be used to treat these respective tumor types only in clinical trials, and 40 (18%), 60 (26%), and 67 (29%) respondents, respectively, indicated that robotic surgery should not be used to treat these tumors. 56 (25%) respondents indicated that T2 tumors should or could be treated with robotic surgery, 54 (24%) considered this application to be appropriate only in clinical trials, and 118 (51%) indicated that robotic surgery should not be used to treat T2 tumors. No respondent believed that robotic surgery should be used to treat T3/4 tumors, 21 (9%) respondents indicated that this technique could be used for these tumors, 35 (15%) accepted such use only in clinical trials, and 172 (76%) indicated that robotic surgery should not be used in such cases (Table 3).

**Table 3:** Participants opinion about robotic treatment of ovarian cancer

	*N* (%)
What is your personal opinion on robotic treatment of ovarian cancer (depending on T stage of disease)?	
T1a tumors	
Should be treated via robotic surgery	40 (18%)
Can be treated via robotic surgery	96 (42%)
Should only be treated via robotic surgery in clinical trials	52 (22%)
Should not be treated via robotic surgery	40 (18%)
T1b tumors	
Should be treated via robotic surgery	32 (14%)
Can be treated via robotic surgery	76 (33%)
Should only be treated via robotic surgery in clinical trials	60 (26%)
Should not be treated via robotic surgery	60 (26%)
T1c tumors	
Should be treated via robotic surgery	32 (14%)
Can be treated via robotic surgery	72 (32%)
Should only be treated via robotic surgery in clinical trials	57 (25%)
Should not be treated via robotic surgery	67 (29%)
T2 tumors	
Should be treated via robotic surgery	22 (10%)
Can be treated via robotic surgery	34 (15%)
Should only be treated via robotic surgery in clinical trials	54 (24%)
Should not be treated via robotic surgery	118 (51%)
T3/4 tumors	
Should be treated via robotic surgery	0 (0%)
Can be treated via robotic surgery	21 (9%)
Should only be treated via robotic surgery clinical trials	35 (15%)
Should not be treated via robotic surgery	172 (76%)

### Perspectives on available data and clinical trial participation

One hundred sixty-two (71%) respondents considered the currently available data on the use of robotic surgery to treat ovarian cancer to be insufficient, and 66 (29%) respondents could not provide any information. 98 (43%) and 50 (22%) of respondents respectively felt that further research on the use of robotic surgery to treat T1/2 and T3/4 ovarian cancer was needed. 95 (42%) respondents indicated that they were willing to participate in clinical trials investigating the robotic surgical treatment of T1/2 carcinomas; as optimal study designs, 128 (56%) respondents suggested a prospective randomized study, 68 (30%) suggested a meta-analysis, and 32 (14%) suggested a retrospective study. 50 (22%) respondents indicated that they would not participate in clinical trials on the use of robotic surgery in the treatment of T3/4 ovarian tumors, 53 (23%) respondents indicated that they would participate in such trials; and 50 (22%) could not provide any information; for research on this topic, 108 (47%) respondents suggested a prospective randomized study, 66 (29%) a meta-analysis, and 54 (24%) a retrospective study, respectively (Table 4).

**Table 4:** Participants opinion about the currently available data on robotic treatment of ovarian cancer

	*N* (%)
How would you assess current data on robotic treatment of ovarian cancer?	
Sufficient	0 (0%)
Insufficient	162 (71%)
I can’t tell	66 (29%)
Do you think robotic treatment for early stage ovarian cancer (T1/2) needs further evaluation?	
Yes	98 (43%)
No	77 (34%)
I can’t tell	53 (23%)
If yes, what kind of study would you suggest?	
Retrospective study	32 (14%)
Meta-analysis	68 (30%)
Prospective randomized controlled trial	128 (56%)
Would you take part in a clinical trial assessing robotic treatment of early stage (T1/2) ovarian cancer?	
Yes	95 (42%)
No	89 (39%)
I can’t tell	44 (19%)
Do you think robotic treatment of advanced ovarian cancer (T3/4) needs further evuluation?	
Yes	50 (22%)
No	128 (56%)
I can’t tell	50 (22%)
If yes, what kind of study would you suggest?	
Retrospective study	54 (24%)
Meta-analysis	66 (29%)
Prospective randomized controlled trial	108 (47%)
Would you take part in a clinical trial assessing robotic treatment of early stage (T3/4) ovarian cancer?	
Yes	53 (23%)
No	124 (55%)
I can’t tell	50 (22%)

### Robotic surgery for borderline ovarian tumors

In total, 158 (69%) respondents stated that borderline ovarian tumors would not be treated with robotic surgery in their practices, 45 (20%) respondents treated such tumors with robotic surgery at their facilities and 25 (11%) respondents reported the use of such treatment in clinical trials. 160 (70%) respondents stated that borderline ovarian tumors should or could be treated with robotic surgery, and 34 (15%) respondents each did and did not favor such treatment within the framework of clinical studies. 126 (55%) respondents indicated that the treatment of borderline ovarian tumors by robotic surgery required further evaluation, 49 (22%) were opposed to new studies on the subject, and 53 (23%) could not provide any information. Similar numbers of respondents reported that they would [*n* = 93 (41%)] and would not [*n* = 102 (45%)] participate in clinical trials on robotic surgical therapy for borderline ovarian tumors; as study designs, 34 (15%) respondents suggested a retrospective study, 54 (24%) a meta-analysis, and 140 (61%) a prospective randomized controlled study, respectively (Table 5).

**Table 5:** Participants opinion and current practice at their institution regarding robotic treatment of ovarian borderline tumors

	*N* (%)
What is your personal opinion on robotic treatment of borderline tumors	
Should be treated via robotic surgery	70 (31%)
Can be treated via robotic surgery	90 (39%)
Should only be treated via robotic surgery in clinical trials	34 (15%)
Should not be treated via robotic surgery	34 (15%)
What is the current practice at your institution (depending on T stage of disease)?	
Borderline tumors	
Are treated via robotic surgery	45 (20%)
Are only treated as part of clinical trials via robotic surgery	25 (11%)
Are not treated via robotic surgery	158 (69%)
Do you think robotic treamtent for ovarian borderline tumors needs further evaluation?	
Yes	126 (55%)
No	49 (22%)
I can’t tell	53 (23%)
If yes, what kind of study would you suggest?	
Retrospective study	34 (15%)
Meta-analysis	54 (24%)
Prospective randomized controlled trial	140 (61%)
Would you take part in a clinical trial assessing robotic treatment of ovarian borderline tumors?	
Yes	93 (41%)
No	102 (45%)
I can’t tell	33 (14%)

## Discussion

The aim of the present study was to examine the opinion of the AGE members regarding the use of robotic surgery in the treatment of ovarian neoplasia and to survey the current application of robotic surgery in their respective facilities. This survey showed that the use of robotic surgery in the treatment of ovarian tumors was not prevalent in the facilities of responding AGE members; 28% of respondents indicated that T1a tumors were treated by robotic surgery, including in clinical studies, and the proportion declined with increasing tumor stage to 5% for T3/4 tumors in clinical trials alone. Thirty-one percent of respondents reported the use of robotic surgery to treat borderline ovarian tumors, of which 11% reported this application only in clinical trials. In contrast, AGE members favored the use of robotic surgery, especially for T1a–c tumors (60% for T1a and declining thereafter) and borderline tumors (70%).

These opinions coincide with findings from retrospective case–control studies conducted in Italy and China, which confirmed that the adequacy and reliability of robotic surgery for the treatment of early-stage ovarian cancer, in terms of surgical outcome and oncological safety, were equivalent to those of conventional laparotomy, although sample sizes were small (7–33 patients) [16–18]. Other groups have proposed the use of robotic surgery at least for the staging of early ovarian cancer in selected patient groups, and as a possible alternative to laparoscopy when performed by gynecologists with surgical experience [19, 20].

The literature contains contradictory information about differences in the outcomes of robotic surgery and conventional laparoscopy. In a prospective randomized study of endometrial cancer treatment, Mäenpää et al. [21] showed that surgical outcomes were equivalent, although robot-assisted surgery reduced the operating time and rate of conversion to laparotomy relative to conventional laparoscopy. In contrast, El Khouly et al. [22] observed equivalent technical efficiency of the two techniques, but shorter operating times for conventional laparoscopy performed to remove adnexal findings the same technical efficiency.

Nezhat et al. [23] compared perioperative outcome and complication rates between laparotomy, laparoscopy and robotic surgery in the treatment of ovarian, tube and peritoneal cancer in their study. In contrast to the opinion of AGE members obtained in the present study, Nezhat et al., concluded in their retrospective study that laparoscopy and robotic surgery are not inferior to laparotomy in early and advanced stages with regard to perioperative outcome, and appear therefore as an acceptable alternative in the therapy of selected patients. In agreement with AGE members, other authors have expressed clear reservations about the use of robotic surgery for the treatment of T3/4 tumors [16–18].

Divergent perspectives on the safety of robotic surgery in the treatment of ovarian neoplasia, about which up to 30% of participants in the present study expressed concern, have been discussed in the literature. For example, some groups have argued that tumor capsule rupture is relevant for overall survival, whereas others have stated that intraoperative rupture does not shorten the progression-free survival time [24–26]. In a review, Minig et al. [20] expressed no concern about the use of robotic surgery in terms of inaccurate peritoneal staging, at least in the early stages of ovarian cancer, as long as the staging is performed by experienced surgeons in appropriate centers; they noted that further studies of the application of robotic surgery in advanced ovarian cancer staging are needed. With regard to intra-abdominal tumor cell carryover, animal experiments have shown the increased production of interleukins and growth factors in the presence of pneumoperitoneum, and in vitro experiments have shown increased ovarian carcinoma cell line growth rates after exposure to carbon dioxide [27, 28]. However, in vivo studies showed no increase in the frequency of recurrence after MIS compared with open surgery [29–31]. Seror et al. [32] found no metastasis in trocar site areas at a median of 504 days after the robotic surgical treatment of ovarian, endometrial, and cervical carcinomas. Another group reported a low rate (1.41%) of port-site metastasis after the use of robotic surgery to treat gynecological malignancies, which coincided with the rate of metastasis in the puncture canal after traditional laparoscopy (1.96%) [9, 33]. These rates are also comparable to those of recurrence in the scar area after conventional laparotomy [34]. Whether the instruments used in robotic surgery modify the risk of metastasis at trocar puncture sites has not been clarified sufficiently; further clinical studies are required [33].

The discrepancy between the currently limited use of robotic surgery in the treatment of ovarian malignancies (even T1a tumors) and AGE members' opinions about the applicability of this technique can be attributed to the limitations of robotic surgery in general, which have hampered the spread of this technique in Germany. The main limitations are the greater cost of equipment acquisition and maintenance relative to that for conventional laparoscopy, and the associated lack of equipment available for treatment and research [35]. In addition, medical staff and surgeons require special training before they can routinely handle surgical robots [36]. According to the *Deutsche Ärztezeitung*, 135 DaVinci® systems (Intuitive Surgical, Inc., Sunnyvale, CA, USA) were in clinical use in Germany in 2019, although (co-)use by gynecologists was not reported specifically [37]. Traditionally, urology is considered to be a pioneering discipline in the use of robotic surgery [38]. The members of AGE, a specialist society that concentrates on conventional laparoscopic operations and is composed mainly of surgeons versed in this technique, may lack robotic surgical expertise, contributing to their reluctance to use robotic surgery.

Advantages of robotic surgery over conventional laparotomy that are shared with those of conventional laparoscopy are reduced blood loss, operation time, postoperative pain, and hospitalization [39]. Advantages of robotic surgery over conventional laparoscopy are the more ergonomic working position and reduced fatigue rate for surgeons, free mobility of the instruments in seven degrees of freedom, and digital networking of surgical robots [40].

The current S3 guidelines for ovarian malignancies do not mention robotic surgery, and recommend laparoscopic staging only in the research context, due to the lack of sufficient high-quality evidence [10]. This coincides with AGE members’ perspectives that further studies of robotic surgery in the gynecological context are needed, and their willingness to participate in such studies. It also corresponds to the state of the literature. For example, Yim et al. [41] found in a review that the majority of studies of the use of robotic surgery for the treatment of gynecological malignancies were retrospective or descriptive. Thus, prospective randomized studies of the application of this technique, at least to T1/2 ovarian tumors, appear to be needed [42].

The United States’ National Comprehensive Cancer Network guidelines mention only the use of conventional laparoscopy by experienced surgeons for the primary treatment of T1a–c tumors; they recommend the use of a minimally invasive access route for intermittent tumor debulking, with possible intraoperative conversion to open surgery via an abdominal incision [43]. This less cautious approach to the use of MIS for ovarian cancer treatment, which is based on greater experience and more widespread practice, is also reflected in the current literature from the United States. Two retrospective studies showed that robotic surgery was equivalent to conventional laparotomy. Feuer et al. [44] described equivalent tumor reduction (73% and 50%) and 1-year recurrence and survival rates (97% and 90%), regardless of tumor stage. Magrina et al. [45] observed no difference in overall survival after conventional laparoscopy (75%), robotic surgery (67%), and laparotomy (66%). However, these studies were conducted with very small patient cohorts (*n* = 89 and 75, respectively) and only 1-year follow-up periods.

In surveys, members of the Society of Gynecologic Oncology (SGO) reported positive attitudes toward and current implementation of robotic surgery in practice [46, 47]. The data revealed the increased use of robotic surgery to treat uterine malignancies and ovarian cancer (especially in the early stages). In one survey, 66% of respondents wished to increase the use of robotic surgery in the future [46]. In the other survey, 97% of respondents reported the use of robotic surgery, especially for the treatment of cervical and endometrial cancers [47]. In agreement with the AGE respondents, the SGO respondents favored additional research on the use of robotic surgery to treat ovarian tumors [46]. The more widespread use of robotic surgery in the Anglo-American region than in Germany may be due, among other factors, to differences in the remuneration system. In 2019, for example, a large portion of radical hysterectomies was performed robotically [37].

Limitations of the present study include the low response rate and the selection bias generated by surveying exclusively (laparoscopy-favoring) AGE members. Future surveys on the use of robotic surgery for ovarian tumor treatment should be conducted with larger and more diverse groups. Another limiting factor was the lack of reliable data on surgical robot use in German gynecological hospitals, which prevented, for example, determination of the ratio of respondents using robotic surgery to the number of robots present in their facilities. The collection of precise data on the quantity and distribution of surgical robots in Germany is crucial for subsequent surveys. A limitation of the present work is the imprecision of the question about the concerns regarding the oncologic safety of robotic surgery as, this question related to MIC in general and did not distinguish between robotic and laparoscopic surgery. Interpreting the results, we must consider the fact that the survey was carried out in 2015. Given the rapid progress in the surgical field, especially in terms of the use of MIS, a limitation of the present study could be the modified opinion of the AGE members regarding the use of robotic surgery in 2020.

## Conclusion

The present survey should give an impression of the current tendencies of German AGE members (albeit in a laparoscopic oriented group) and the current practice in German hospitals. Prospective randomized studies on the therapy of T1/2 ovarian tumors as well as borderline tumors of the ovary should be implemented according to the results of this survey.

Giving the current evidence, robotic surgery for the treatment of ovarian malignancies should only be performed on selected patients within clinical studies.

## Data Availability

The dataset used and analyzed during the current study is available from the corresponding author on reasonable request.
