# Correction to: Survey of current practices and opinions of German Society of Gynecologic Endoscopy members regarding the treatment of ovarian neoplasia by robotic surgery

**DOI:** 10.1007/s00404-021-06136-1

**Published:** 2021-07-17

**Authors:** J. S. M. Zimmermann, J. C. Radosa, M. P. Radosa, P. Sklavounos, P. A. Schweitzer, E. F. Solomayer

**Affiliations:** 1 Department of Gynecology and Obstetrics, Saarland University Hospital, Kirrbergerstraße 100, 66421 Homburg, Saar Germany; 2 Department of Gynecology and Obstetrics, Klinikum Bremen Nord, Bremen, Germany

## Correction to: Archives of Gynecology and Obstetrics (2021) 303:1305–1313 10.1007/s00404-020-05876-w

The article “Survey of current practices and opinions of German Society of Gynecologic Endoscopy members regarding the treatment of ovarian neoplasia by robotic surgery” written by J. S. M. Zimmermann, J. C. Radosa, M. P. Radosa, P. Sklavounos, P. A. Schweitzer and E. F. Solomayer was originally published electronically on the publisher’s internet portal on November 17, 2020 without open access. With the author(s)’ decision to opt for Open Choice the copyright of the article changed to © The Author(s) 2020 and the article is forthwith distributed under a Creative Commons Attribution 4.0 International License, which permits use, sharing, adaptation, distribution and reproduction in any medium or format, as long as you give appropriate credit to the original author(s) and the source, provide a link to the Creative Commons licence, and indicate if changes were made. The images or other third-party material in this article are included in the article’s Creative Commons licence, unless indicated otherwise in a credit line to the material. If material is not included in the article’s Creative Commons licence and your intended use is not permitted by statutory regulation or exceeds the permitted use, you will need to obtain permission directly from the copyright holder. To view a copy of this licence, visit http://creativecommons.org/licenses/by/4.0/. Open Access funding enabled and organized by Projekt DEAL.

The original article has been updated.

